# Chinese herbal bath therapy for the treatment of uremic pruritus: meta-analysis of randomized controlled trials

**DOI:** 10.1186/s12906-019-2513-9

**Published:** 2019-05-10

**Authors:** Wenxuan Xue, Yanhua Zhao, Mengyun Yuan, Zhiqiang Zhao

**Affiliations:** The First College of Clinical Medicine, Nanjing University of Chinese Medicine, 138 Xianlin Avenue, Qixia District, Nanjing City, Jiangsu Province China

**Keywords:** Bath, Uremic pruritus, ESRD, Haemodialysis, Traditional Chinese medicine

## Abstract

**Background:**

Chinese herbal bath therapy (CHBT) is a traditional external therapy that has been used for the treatment of uremic pruritus (UP) in China. We conducted a meta-analysis to evaluate the efficacy and safety of CHBT for UP.

**Methods:**

We searched seven databases for studies published since database inception to September 1, 2018. Randomized trials evaluating CHBT for UP were collected. The therapeutic effects of CHBT were measured by the pruritus level (via the visual analogue scale (VAS) or the symptom score scale) and the total effective rate. We combined studies using mean difference (MD) for continuous outcomes and using risk ratio for dichotomous data, both with 95% confidence intervals. RevMan V.5.3 software was used to assess the data reported and perform the meta-analysis.

**Results:**

Seventeen articles including 970 patients were identified. All participants were haemodialysis (HD) patients. CHBT is administered by immersing the whole body in a prepared herbal water bath. On average, an herbal bath prescription included 11 Chinese herbs. The mean treatment duration was 4.7 weeks. Compared with basic treatment (HD or haemoperfusion (HP)) and adding a control of sham CHBT, clear hot water bath, or calamine lotion, CHBT plus basic treatment reduced the VAS score (MD = − 2.38; 95% confidence intervals [CI], − 3.02 to − 1.74; *P* < 0.00001) and the symptom score (MD = − 8.42; 95% confidence intervals [CI], − 12.47 to − 4.36; *P* < 0.00001) and had a higher total effectiveness rate (risk ratio [RR] = 1.46; 95% CI, 1.31 to 1.63; *P* < 0.00001).

**Conclusions:**

In conclusion, CHBT could be a complementary therapy for improving pruritic symptoms in uraemia patients. More rigorously designed, multicentre, prospective RCTs are warranted to further identify the efficacy and safety of CHBT.

**Trial registration:**

Systematic review registration: [PROSPERO registration: CRD42018108506].

**Electronic supplementary material:**

The online version of this article (10.1186/s12906-019-2513-9) contains supplementary material, which is available to authorized users.

## Background

Uremic pruritus (UP) is one of the most common and troublesome symptoms in patients with chronic renal failure and occurs in 46% of haemodialysis patients [1–3]. UP causes great distress to patients and affects their mood and sleep quality. In addition, UP has an independent relationship with mortality, which is an urgent problem to solve [4–6].

The pathophysiology of UP is incompletely understood and likely multifactorial. Skin or nerve inflammation [6, 7], subclinical or overt uremic neuropathy in the context of chronic systemic inflammation associated with renal failure, or increased μ-opioid receptor activity due to kidney failure have all been related [3, 8, 9].

UP is often difficult to eradicate, although the symptoms can usually be mitigated. Current medications including gabapentin (a modulator of excitatory neurotransmitters) [10], capsaicin (a mediator of substance P release) [11], and cromolyn sodium (a mast cell stabilizer) [12, 13], have been suggested, but no specific, effective therapies are currently available for uremic pruritus [1, 3].

As an ancient therapeutic method, Chinese herbal bath therapy (CHBT) has been used for thousands of years in China [14, 15]. In the early published Chinese medical works, such as “Prescriptions for fifty-two diseases” (202 B.C.-9 A.D.) and “Treatise on Cold Pathogenic and Miscellaneous Diseases” (200 A.D.- 210 A.D.), herbal bath therapy was frequently used [15]. Importantly, herbal bath therapy is still very popular in Asian countries today and is widely used to treat skin diseases, osteoarthritis diseases and some internal diseases, of which pruritus is especially responsive [16]. While traditional Chinese medicine doctors use herbal baths to relieve itching in patients with uraemia, the mechanism of the effect is not particularly clear [14, 17].

To evaluate the efficacy and effectiveness of Chinese herbal bath therapy scientifically, we reviewed the medical literature comprehensively and conducted a meta-analysis of randomized controlled trials of herbal bath therapy focusing on the treatment of uremic pruritus.

## Methods

### Protocol and registration

This study was registered in the Preferred Reporting Items for Systematic Review and Meta-analyses Statement (PRISMA) under the registration number: CRD42018108506 (available from: http://www.crd.york.ac.uk/PROSPERO/display_record.php? ID = CRD42018108506).

### Search strategy

We searched PubMed, Cochrane Library, Embase, Chinese National Knowledge Infrastructure (CNKI), Wanfang Databases, Chinese Science and Technology Periodical Database (VIP) and Chinese Biomedical Database (CBM) from their inception to September 1, 2018. The search strategy for PubMed was: Search (((((((((((((((((((((((((((((((“Kidney Failure, Chronic”[Mesh]) OR chronic kidney failure) OR chronic kidney disease) OR chronic kidney insufficiency) OR chronic nephropathy) OR chronic renal disease) OR chronic renal failure) OR kidney chronic failure) OR kidney disease, chronic) OR kidney failure, chronic) OR kidney function, chronic disease) OR renal insufficiency, chronic) OR End-Stage Kidney Disease) OR Disease, End-Stage Kidney) OR End Stage Kidney Disease) OR Kidney Disease, End-Stage) OR End-Stage Renal Disease) OR Disease, End-Stage Renal) OR End Stage Renal Disease) OR Renal Disease, End-Stage) OR Renal Disease, End Stage) OR Renal Failure, End-Stage) OR End-Stage Renal Failure) OR Renal Failure, End Stage) OR Renal Failure, Chronic) OR ESRD)) OR ((((((((((((((((((((((((((((((((((((“Renal Dialysis”[Mesh]) OR Dialyses, Renal) OR Renal Dialyses) OR Dialysis, Renal) OR Hemodialyses) OR Dialysis, Extracorporeal) OR Dialyses, Extracorporeal) OR Extracorporeal Dialyses) OR Extracorporeal Dialysis) OR hemodialysis) OR blood dialysis) OR chronic haemodialysis) OR chronic hemodialysis) OR chronic intermittent haemodialysis) OR chronic intermittent hemodialysis) OR dialysis center) OR dialysis, blood) OR extracorporeal blood cleansing) OR haemodialysis) OR haemodialysis center) OR haemodialysis centre) OR haemodialysis department) OR haemodialysis unit) OR haemodialysis units, hospital) OR hemodialyse) OR hemodialysis center) OR hemodialysis department) OR hemodialysis unit) OR hemodialysis units, hospital) OR hemorenodialysis) OR hemotrialysate) OR intermittent chronic haemodialysis) OR intermittent chronic hemodialysis) OR intermittent haemodialysis) OR intermittent hemodialysis) OR kidney dialysis)) OR ((((((((“Peritoneal Dialysis”[Mesh]) OR Dialyses, Peritoneal) OR Dialysis, Peritoneal) OR Peritoneal Dialyses) OR intermittent peritoneal dialysis) OR peritoneal dialysis, intermittent) OR peritoneum dialysis) OR peritoneum inigation))) AND ((((“Pruritus”[Mesh]) OR Pruritis) OR Itching) OR itch)) AND ((((((((((“Baths”[Mesh]) OR Bath) OR bathing) OR bathroom) OR baths) OR hydroelectric bath) OR water bath) OR waterbath) OR wash) OR washing). A similar strategy was applied to the Cochrane Library and Embase. These search terms were adapted and used in the Chinese databases with no limitations.

### Eligibility criteria

Studies were included for analysis if they met the following criteria: (1) The included studies were randomized controlled trials (RCTs) recruiting participants with UP. (2) For the types of interventions, the participants of the treatment groups were given a herbal bath with only the head remaining out of the water. (3) The included studies evaluated the efficacy or effectiveness of CHBT for UP and had clear outcome data, such as total effectiveness rate or symptom scores.

The exclusion criteria were as follows: (1) The CHBT group used other traditional Chinese medicine treatment (OTCMT) methods (The control group combined with OTCMT is not excluded). (2) The details of the trials were unclear, or the outcome data were not complete.

### Selection of studies

The two authors (WX and YZ) conducted the literature screening independently. The titles and abstracts of all records were first filtered for relevance, and then the full text of potentially relevant studies was identified for eligibility. If a disagreement arose, it is resolved through discussion with a third author (MY).

The effects of herbal bath therapy on the clinical symptoms of UP were measured by the pruritus level (via the visual analogue scale (VAS) or the symptom score scale) and the total effective rate. The definitions of the outcome measures are described in detail in Table 1.

**Table 1:** Characteristics of randomized controlled trials of Chinese herbal bath therapy for HP

Source	Study setting (hospital/clinic)	Diagnostic criteria	Disease stage	Sample Size and Included Subject Age (Year)	Intervention (Regimen)	Control (Regimen)	Concomitant Treatments	Uremic Pruritus Measurement Tools
Du, 2009 [19]	hospital	Chinese diagnostic standard	CRF uraemia period	41 HD patients with UP CHBT: 45.7 Control: 44.9	CHBT+HD	CL + HD	Erythropoietin (skin-pop, 3000 IU each time, two or three times a w), treat acidosis, supplement iron, folic acid	Improvement of pruritus
Du et al., 2004 [20]	hospital	Chinese diagnostic standard	CRF uraemia period	29 HD patients with UP CHBT: 45 Control: 46	CHBT+HD	CL + HD	Erythropoietin (skin-pop, 2000 IU each time, two or three times a w), calcium carbonate (1.5 g per pill, 3 times a d), treat acidosis, supplement iron, folic acid	Improvement of pruritus
Gao and Ye, 2012 [21]	hospital	NKF	CKD Stage 5	41 HD patients with UP Mean: 53.9 ± 14.1	CHBT+HD	HP + HD	Medications (dose and frequency: n.r.): erythropoietin, calcium carbonate, calcitriol; healthy diet	DRKS
Guo et al., 2009 [22]	hospital	Chinese diagnostic standard	CRF uraemia period	45 HD patients with UP CHBT: 46.3 ± 12.8 Control: 44.6 ± 13.2	CHBT+HD	CL + HD	Control blood pressure, treat anaemia, maintain water electrolyte and acid-base balance, regulate calcium and phosphorus metabolism, healthy diet	Improvement of pruritus
Jia et al., 2012 [23]	hospital	NKF	CKD Stage 5	33 HD patients with UP CHBT: 52.9 Control: 53.2	CHBT+HD	HP + HD	Treat anaemia, maintain water electrolyte and acid-base balance, healthy diet	DRKS; Improvement of pruritus
Jia, Li and Fei, 2012 [24]	hospital	NKF	CKD Stage 5	30 HD patients with UP Mean: n.r.	CHBT+HD	CL + HD	treat anaemia, maintain water electrolyte and acid-base balance, healthy diet	Improvement of pruritus
Lan, 2017 [25]	hospital	NKF	CKD Stage 5	60 HD patients with UP CHBT: 47.32 ± 11.23 Control: 48.12 ± 10.73	CHBT+HD	CL + HD	Medications (dose and frequency: n.r.): Erythropoietin (skin-pop), calcium carbonate	Improvement of pruritus
Lin, 2014 [26]	hospital	NKF	CKD Stage 5	80 HD patients with UP CHBT: 51.4 ± 5.2 Control: 52.6 ± 3.1	CHBT+HP + HD	HP + HD	Maintain water electrolyte and acid-base balance, treat anaemia	Improvement of pruritus
Shen, 2014 [27]	hospital	NKF	CKD Stage 5	53 HD patients with UP	CHBT+HD	Cetirizine+CL + HD	n.r.	0–10 VAS
Wang et al., 2013 [28]	hospital	NKF	CKD Stage 5	54 HD patients with UP CHBT: 48.9 Control: 46.8	CHBT+HD	HD	maintain water electrolyte and acid-base balance, control blood pressure, diuresis, calcium supplement, anti-infection	100-mm VAS; Improvement of pruritus
Wen, 2007 [29]	hospital	Chinese diagnostic standard	CRF uraemia period	42 HD patients with UP CHBT: 48.2 Control: 48.6	CHBT+HD	CL + HD	Erythropoietin (skin-pop, 3000 IU each time, two or three times a w), treat acidosis, calcium supplement, supplement iron	Improvement of pruritus
Yao, 2015 [30]	hospital	NKF	CKD Stage 5	42 HD patients with UP Mean: 47.5 ± 20.2	CHBT+HD	CL + HD	Treat anaemia, maintain water electrolyte and acid-base balance, calcium supplement	Improvement of pruritus
Yu et al., 2017 [31]	hospital	NKF	CKD Stage 5	30 HD patients with UP Mean: 57.43 ± 10.23	CHBT+HD	CHWB+HD	Control blood pressure and blood sugar, treat anaemia, regulate calcium and phosphorus metabolism	DRKS
Zhang et al., 2014 [32]	hospital	NKF	CKD Stage 5	100 HD patients with UP CHBT: 46 Control: 45	CHBT+HD	CL + HD	Treat anaemia, maintain water electrolyte and acid-base balance, calcium supplement	Improvement of pruritus
Zhang et al., 2012 [33]	hospital	Chinese diagnostic standard	CRF uraemia period	156 HD patients with UP CHBT: 46 Control: 44	CHBT+HD	HD	Erythropoietin (skin-pop, 4000 IU each time, two times a w), calcium carbonate (1.0 g per pill, 3 times a d), supplement iron, folic acid	DRKS; Improvement of pruritus
Zhao, 2011 [34]	hospital	NKF	CKD Stage 5	24 HD patients with UP CHBT: 42.77 ± 13.19 Control: 49.27 ± 9.72	CHBT+HD	Sham CHBT+HD	Treat anaemia and control blood pressure Medications: including haemopoietin, iron, folic acid, calcium channel blockers, ACE inhibitor, angiotensin receptor inhibitor.	10-cm VAS
Zheng, 2016 [35]	hospital	NKF	CKD Stage 5	42 HD patients with UP CHBT: 47.5 ± 20.2 Control: 47.7 ± 20.5	CHBT+HD	HD	Treat anaemia, take calcium and vitamin D supplements, control blood pressure, blood sugar, healthy diet	DRKS

*VAS* visual analogue scale, *ACE inhibitor* angiotensin converting enzyme inhibitor, *w* weeks, *d* days, *n.r.* not reported, *CHWB* Clean hot water bath, *CL* calamine lotion (an external Chinese medicine for relieving itching. The main ingredients are calamine, zinc oxide and glycerine)

Diagnostic criteria:

(i) NKF: National Kidney Foundation. CKD Stage 5 is described as follows: established kidney failure (GFR < 15 ml/min/1.73 m^2^), permanent renal replacement therapy, or end-stage kidney disease

(ii) Chinese diagnostic standard: This is described in the second edition of *Nephrology*, edited by Haiyan Wang. CRF (uraemia period) is described as follows: Scr > 707 μmol/L, BUN> 28.6 mmol/L, accompanied by metabolic acidosis, renal anaemia and other corresponding clinical symptoms

Outcome definition and measurement:

(i) The improvement of pruritus assessment comprises four levels: “cured” (pruritus disappeared or almost disappeared); “significantly improved” (pruritus improved significantly); “improved” (pruritus is relieved); and “not cured” (pruritus did not decrease or even worsened). Total effectiveness rate (%) is determined as the quotient of the number of cured, significantly improved and improved patients divided by the total number of the patients

(ii) DRKS, Dirk R Kuypers score. Main points: (a) relieving the effect of scratching on pruritus: 1-5points (b) pruritic range: 1-3points (c) frequency of itching: 1–5 points (d) disturbed sleep at night due to itching: 2–14 points. The first three items (a, b, c) are evaluated twice a day, once in the morning and once in the afternoon. Twenty-four-hour maximum score: 40 points. Higher scores indicate worse outcomes

### Data extraction

Two authors (WX and YZ) extracted the following data independently using a data extraction table designed prior to initiation of the literature search: publication information, study setting, study population, age, sample size, characteristics of intervention and control, outcome indicators, and relevant indicators of bias risk assessment. The main data are summarized in Table 1 and Table 2.

**Table 2:** Treatment details of included studies

Study	CHBT group intervention		Control group intervention		Duration
	frequency of HD or HP	CHBT	frequency of HD or HP	other methods	
Du, 2009 [19]	HD: 2 or 3 times a w, 4 h each time	10 herbs: 30–100 g each, bathed for 30–40 min, once a d	HD: same as the CHBT group	CL: external use, three times a d	30 d
Du et al., 2004 [20]	HD: 2 or 3 times a w, 4 to 5 h each time	9 herbs: 30–150 g each, bathed for 30–60 min, once every two d	HD: same as the CHBT group	CL: external use, 3 times a d	30 d
Gao and Ye, 2012 [21]	HD: 9 to 12 h a w	10 herbs: 15–50 g each; WT:40-45 °C; bathed for 20–30 min; three times a w	HD: same as the CHBT group HP: two h each time, once every two w	n.r.	3 m
Guo et al., 2009 [22]	HD: once every 2 to 3 d, 4 h each time	10 herbs: 20–50 g each, WT: 40-45 °C; bathed for 30–60 min, once a d	HD: same as the CHBT group	CL: external use, two or three times a d	2 w
Jia et al., 2012 [23]	HD: 2 or 3 times a w; HP: once every two w, 2 h each time	11 herbs: 15–50 g each; WT: 40-43 °C; bathed for 30 min; once a d	HD, HP: same as the CHBT group	n.r.	8 w
Jia, Li and Fei, 2012 [24]	HD: regular.	11 herbs: 15–50 g each; WT: 40-43 °C; bathed for 30 min; once a d	HD: same as the CHBT group	external use, three or four times a d	4 w
Lan, 2017 [25]	HD: 2 or 3 times a w, 4 h each time	10 herbs: 20–50 g each, bathed for 30–60 min, once every two d	HD: same as the CHBT group	CL: external use, 2 or 3 times a d	30 d
Lin, 2014 [26]	HD: 2 or 3 times a w; HP: 2 times a m	11 herbs: 15–50 g each, WT: approximately 42 °C, bathed for 30 min, once a d	HD: 2 or 3 times a w; HP: 2 times a m	n.r.	2 m
Shen, 2014 [27]	HD: regular.	14 herbs, bathed for 30–40 min, once a d	HD: same as the CHBT group	CL: external use Cetirizine: take orally Dose and frequency: n.r.	2 w
Wang et al., 2013 [28]	HD: regular.	11 herbs: 5–30 g each, bathed for 30 min, once a d	HD: same as the CHBT group	n.r.	4 w
Wen, 2007 [29]	HD: 2 or 3 times a w, 4 to 5 h each time	9 herbs: 30–200 g each, bathed for 30–60 min, once every two d	HD: same as the CHBT group	CL: external use, 3 times a d	20 d
Yao, 2015 [30]	HD: regular.	6 kinds of Chinese medicine granules: 10–30 g each, WT: 40-45 °C, bathed for 15–30 min, once a d	HD: same as the CHBT group	CL: external use, two or three times a d	2 w
Yu et al.,2017 [31]	HD: 3 times a w, 4 h each time	10 herbs: 50–100 g each, hot water, bathed for 30 min, once a d	HD: same as the CHBT group	Clear hot water bath: 30 min at a time	2 w
Zhang et al., 2014 [32]	HD: regular.	6 kinds of Chinese medicine granules, WT: 40-45 °C, bathed for 15–30 min, once a d	HD: same as the CHBT group	CL: external use, two or three times a d	2 w
Zhang et al., 2012 [33]	HD: regular.	13 herbs, warm water, once a d	HD: same as the CHBT group	n.r.	2 w
Zhao, 2011 [34]	HD: regular.	22 herbs, WT: 37-40 °C, bathed for 30–40 min, once a d	HD: same as the CHBT group	Sham CHBT: water with the same colour and smell as the experimental group	2 w
Zheng, 2016 [35]	HD: 2 or 3 times a w, 4 h each time	9 herbs, bathed for 30 min, once a d	HD: same as the CHBT group	n.r.	3 m

*WT* water temperature, *m* months, *w* weeks, *d* days, *h* hours, *n.r.* not reported

### Statistical analysis

We utilized Review Manager (Version 5.3, The Cochrane Collaboration, 2014) for data synthesis and analysis. For continuous outcomes, we analysed studies using mean difference (MD) with a 95% confidence interval. MD was calculated by subtracting after from before measurements, and standard deviation (SD) for change was estimated by the given SD values before and after treatment. In addition, for dichotomous data, we combined studies using a risk ratio to compare intervention and control groups with a 95% confidence interval. As the outcomes of this meta-analysis, VAS and Dirk R Kuypers score (DRKS) were presented as MD, while a total effectiveness rate was presented as RR. Due to significant clinical heterogeneity, we used a random-effects model for pooling. Heterogeneity was evaluated statistically using the Cochran Q statistic and the *I*^2^ index. We used funnel plots to evaluate the potential publication bias.

### Methodological quality

Two authors (WX and MY) independently appraised the methodological quality of the included studies according to the Cochrane Collaboration’s risk of bias tool [18]. Any discrepancy between the investigators was resolved by the third author (YZ). The following 7 aspects were assessed: (1) random sequence generation; (2) allocation concealment; (3) patient blinding; (4) assessor blinding; (5) incomplete outcome data; (6) selective reporting; and (7) other sources of bias. The evaluation results were given a ranking of low risk, unclear risk, or high risk according to the above criteria.

## Results

### Search results

**Fig. 1:**
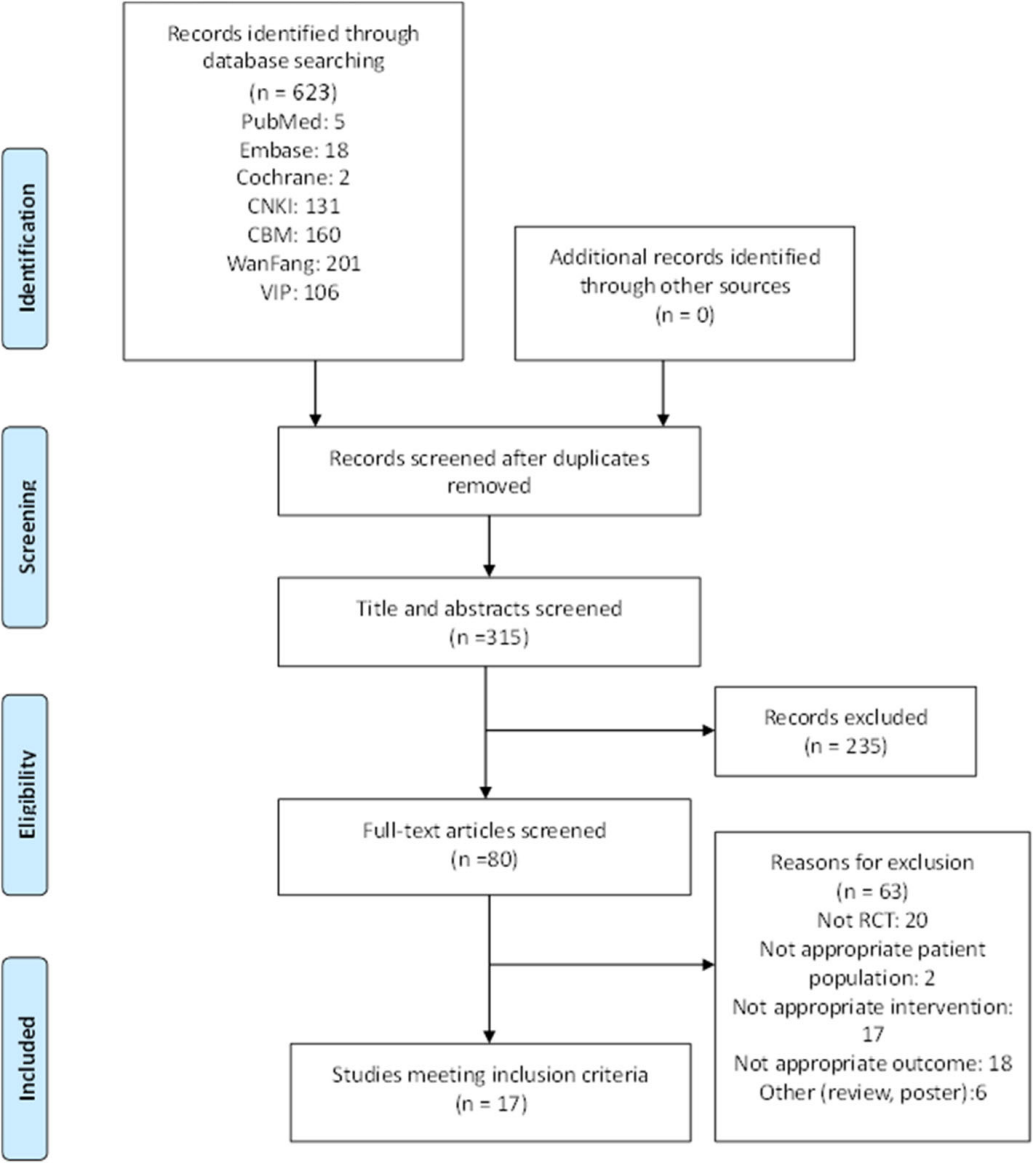
Study selection flow chart

We searched 623 articles from 7 electronic databases according to the search strategy. A total of 308 duplicate articles were excluded. After initially screening the titles and abstracts, 235 articles were excluded because they did not fit the inclusion criteria. We retrieved 80 full-text studies for further identification, and 63 articles were removed with reasons. Finally, 17 eligible RCTs [19–35] (For links to the articles, see Additional file 1) and 970 participants were included in the meta-analysis (Fig. 1).

### Study characteristics

The basic characteristics of the 17 RCTs are shown in Table 1, and the details of each research scheme are shown in Table 2. All of the RCTs were conducted and published in China. A total of 970 patients with UP were enrolled, with the sample size ranging from 24 to 156. All participants were on haemodialysis and were undergoing basic treatment, such as controlling blood pressure, treating anaemia, or maintaining water electrolyte and acid-base balance. All of the patients were adults over the age of 18. The mean treatment duration was 4.7 weeks.

On average, an herbal bath prescription in the intervention groups included 11 Chinese herbs, ranging from 6 to 22. In these prescriptions, two [30, 32] used granules of traditional Chinese medicine as raw materials to replace herbs. The relationship among English names, Latin names and Chinese PinYin names of the 19 most frequently used Chinese herbs are listed in Table 3. Eight Chinese herbs (Fructus Kochiae, Lightyellow Sophora Root, Densefruit Pittany Root-bark, Cicada Slough, Common Cnidium Fruit, Chinese Honeylocust Spine, Puncturervine Caltrop Fruit, and Tuber Fleeceflower Root) were described to relieve pruritus. Five Chinese herbs (Chinese Angelica, Manchurian Wildginger, Szechuan Lovage Rhizome, Suberect Spatholobus Stem, and Dan-shen root) were described to activate blood circulation. Four Chinese herbs (Chinese Ephedrs Herb, Fineleaf Schizonepeta Herb, Angelica Dahurica, and Divaricate Saposhniovia Root) were described to stimulate sweat. Two Chinese herbs, Rhubarb Root and Glabrous Greenbrier Rhizome, were described to promote detoxification [36–38]. The control groups used modern basic medical treatment, some of which were combined with sham CHBT, clean hot water bath, or calamine lotion. Treatment duration in the RCTs ranged from 2 weeks to 3 months, and outcomes were evaluated at the end of treatment.

**Table 3:** The 19 most frequently used flavours of Chinese herbal medicine in 17 bath prescriptions

English name	Latin name	Chinese Pinyin name	Frequency of usage
Pruritus relief			
Fructus Kochiae	Fructus Kochiae Scopariae	Difuzi	15
Lightyellow Sophora Root	Radix Sophorae Flavescentis	Kushen	13
Densefruit Pittany Root-bark	Cortex Dictamni	Baixianpi	13
Cicada Slough	Periostracum Cicadae	Chantui	12
Common Cnidium Fruit	Fructus Cnidii	Shechuangzi	4
Chinese Honeylocust Spine	Spina Gleditsiae	Zaojiaoci	3
Puncturervine Caltrop Fruit	Fructus Tribuli Terrestris	Baijili	3
Tuber Fleeceflower Root	Radix Polygoni Multiflori	Heshouwu	3
Activating blood circulation			
Chinese Angelica	Radix Angelicae Sinensis	Danggui	12
Manchurian Wildginger	Herba Asari	Xixin	12
Szechuan Lovage Rhizome	Rhizoma Chuanxiong	Chuanxiong	11
Suberect Spatholobus Stem	Caulis Spatholobi	Jixueteng	5
Dan-shen root	Radix Salviae Miltiorrhizae	Danshen	4
Stimulating secretion of sweat			
Chinese Ephedrs Herb	*Ephedra sinica* Stapf	Mahuang	7
Fineleaf Schizonepeta Herb	Herba Schizonepetae	Jingjie	8
Angelica Dahurica	Radix Angelicae Dahuricae	Baizhi	6
Divaricate Saposhniovia Root	Radix Saposhnikoviae	Fangfeng	4
Promoting detoxification			
Rhubarb Root and Rhizome	Rheum Officinale Baill	Dahuang	7

### Meta-analysis

In the 17 eligible RCTs, three trials measured pruritus via the VAS score and five trials used the Dirk R Kuypers score, whereas 12 trials assessed clinical improvement by total effectiveness rate. Three trials measured and evaluated pruritus score and total effectiveness rates simultaneously. In addition, in three of these RCTs [28, 33, 35], no antipruritic drugs or HP were used in the control group, primarily reflecting the efficacy of CHBT in the treatment of UP. The 14 other RCTs using antipruritic treatments in the control group were more focused on which treatments were more effective, primarily reflecting the effectiveness of CHBT in the treatment of UP.

#### Continuous data outcomes

**Fig. 2:**
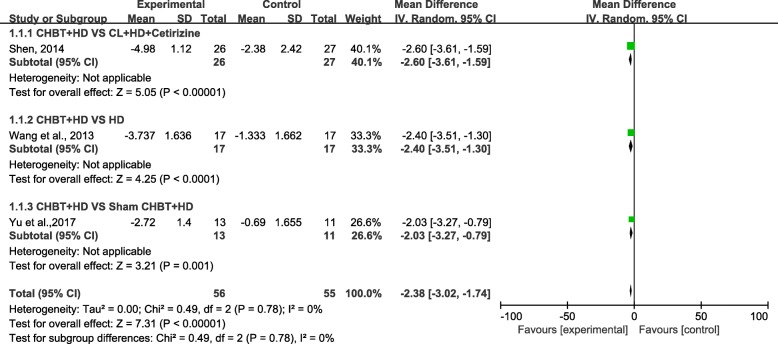
Effect of Chinese herbal bath therapy on pruritus score (VAS)

We unified the VAS of the three trials involving 111 patients into VAS (0–10 points) and conducted statistical analysis according to the data after conversion. The random-effects model was used. Subgroup analysis was performed because of clinical heterogeneity between the different control groups. The comprehensive analysis showed that the pruritus scores of patients in the Chinese herbal bath therapy combined with those of the haemodialysis groups were significantly lower than those in the control groups (MD = − 2.38; 95% confidence intervals [CI], − 3.02 to − 1.74; *P* < 0.00001) after 2–4 weeks of treatment. Subgroup results also support this conclusion. The second subgroup (CHBT+HD VS HD) showed that CHBT may indeed have an effect on the treatment of UP, whereas the other two groups showed better effectiveness than sham CHBT and cetirizine. There was no evidence for statistical heterogeneity across studies (chi-square = 0.49; degree of freedom = 2; *I*^2^ = 0%) (Fig. 2).

**Fig. 3:**
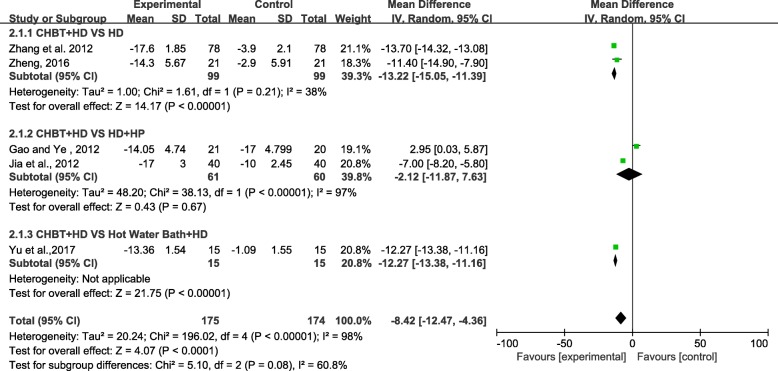
Effect of Chinese herbal bath therapy on pruritus score (DRKS)

Five trials involving 349 patients measured pruritus score based on the Dirk R Kuypers score (0–40 points). We used the random-effects model for statistical analysis. Subgroup analysis was performed according to the different controls. The pooled analysis showed that the pruritus scores of patients in the Chinese herbal bath therapy combined with the haemodialysis or haemoperfusion groups were lower than those in the control groups (MD = − 8.42; 95% confidence intervals [CI], − 12.47 to − 4.36; *P* < 0.00001) after 2 weeks to 3 months of treatment. Three subgroup results also supported this conclusion and reflected the same situation. CHBT may be more effective than HP and a hot water bath. However, the combined analysis has significant statistical heterogeneity (chi-square = 196.02; degree of freedom = 4; *I*^2^ = 98%) (Fig. 3). It was difficult to analyse the causes of heterogeneity due to the small number of studies. The reason maybe that the index value span (0–40 points) is too large, and the scoring process is subjective according to the patients.

As mentioned above, the pruritic score of the patients in Chinese herbal bath therapy was lower than that of the control groups.

#### Total effectiveness rate outcomes

**Fig. 4:**
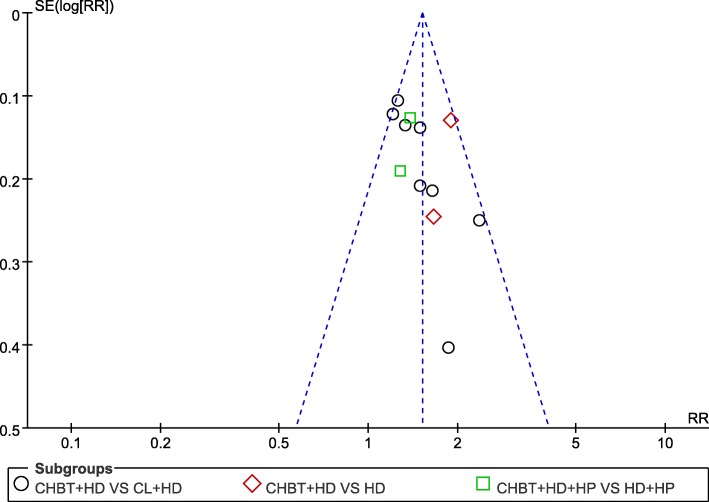
Funnel plot of Chinese herbal bath therapy on overall effectiveness

Twelve trials involving 780 patients reported a comparison of the total effectiveness rate between Chinese herbal bath therapy combined with modern basic medical treatment and control groups using modern basic medical treatment or adding calamine lotion. The results from our random-effects model meta-analysis revealed that Chinese herbal bath therapy combined with modern basic medical treatment improved the clinical effective rates by 46% on average when compared with controls (risk ratio [RR] = 1.46; 95% CI, 1.31 to 1.63; *P* < 0.00001) after 2 weeks to 2 months of treatment, without significant heterogeneity among studies (I^2^ = 27%). An asymmetrical funnel plot of the 12 trials presented potential publication bias as shown in Fig. 4.

**Fig. 5:**
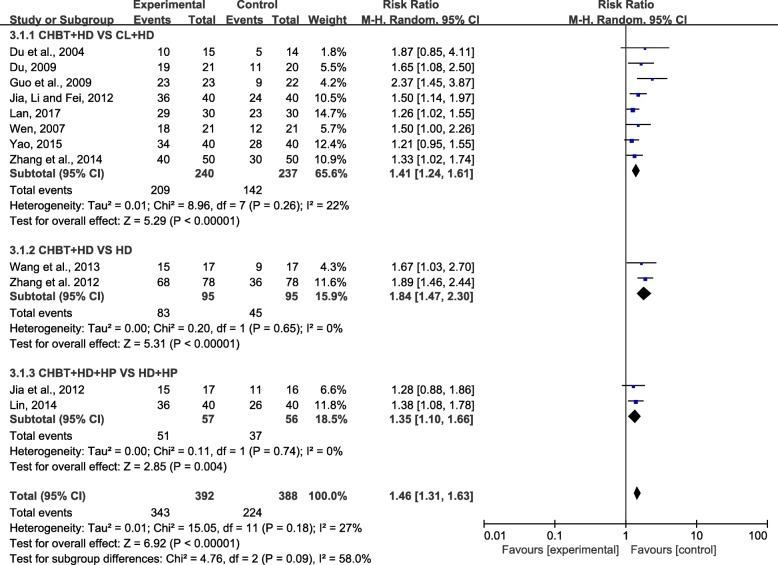
Effect of Chinese herbal bath therapy on overall effectiveness

We performed a further subgroup meta-analysis exploring the improvement of different controls on total effectiveness rate. The results showed that the combined use of Chinese herbal bath therapy and modern basic medical treatment has a better effect when compared with HD (RR = 1.84; 95% CI, 1.47 to 2.30; *P* < 0.00001), HD combined with HP (RR = 1.35; 95% CI, 1.11 to 1.66; *P* < 0.001) or calamine lotion (RR = 1.41; 95% CI, 1.24 to 1.61; *P* < 0.00001) (Fig. 5). As described above, the RR values of subgroups (CHBT+HD VS HD, CHBT+HD + HP VS HD + HP) greater than 1 indicated that CHBT may be effective in treating UP, whereas the RR value of the group (CHBT+HD VS CL + HD) greater than 1 indicated that the CHBT may be more effective than calamine lotion.

Because different dosages of CHBT may lead to different therapeutic effects, we further analysed 8 RCTs in the first subgroup (CHBT+HD VS CL + HD).

**Fig. 6:**
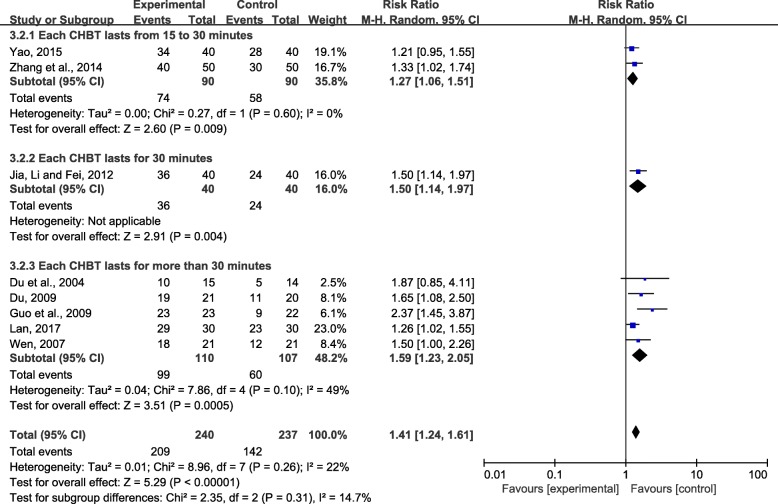
Effect of each CHBT treatment on overall effectiveness

First, we performed further subgroup analysis according to the different duration of each bath. The results of the subgroups were ranked in order of RR value from large to small: each CHBT lasts for more than 30 min (RR = 1.59; 95% CI, 1.23 to 2.05; *P* < 0.001), each CHBT lasts for 30 min (RR = 1.50; 95% CI, 1.14 to 1.97; *P* < 0.01), or each CHBT lasts from 15 to 30 min (RR = 1.27; 95% CI, 1.06 to 1.51; *P* < 0.01) (Fig. 6). The results suggested that appropriate prolonging of each CHBT may be beneficial for the treatment of UP.

**Fig. 7:**
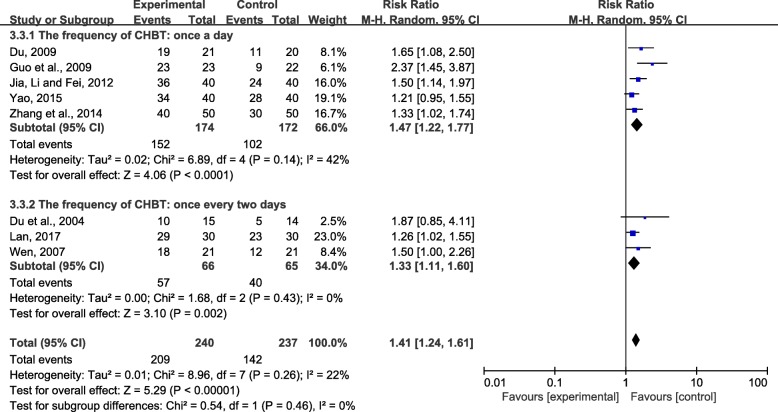
Effect of CHBT frequency on overall effectiveness

Second, we conducted subgroup analysis according to different bath frequency. The results of the two groups were ranked in order of RR value from large to small: once a day (RR = 1.47; 95% CI, 1.22 to 1.77; *P* < 0.001) or once every 2 days (RR = 1.33; 95% CI, 1.11 to 1.60; *P* < 0.01) (Fig. 7). The results suggested that an herbal bath once a day may be more effective than an herbal bath once every other day.

**Fig. 8:**
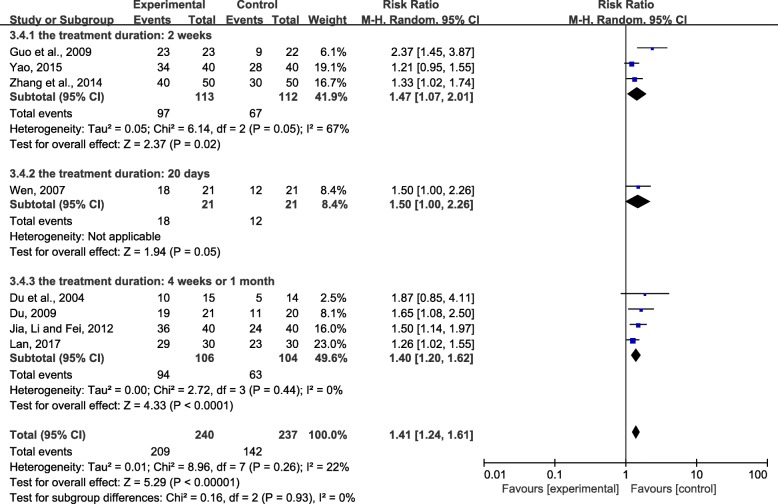
Effect of CHBT duration on overall effectiveness

Third, we conducted a subgroup analysis based on the length of treatment duration. Due to the large *P* values (*P* ≥ 0.05), the second subgroup (the treatment duration was 20 days) was not statistically significant. The results of the other two subgroups were ranked in order of RR value from large to small: treatment duration was 2 weeks (RR = 1.47; 95% CI, 1.07 to 2.01; *P* < 0.05) or treatment duration was 4 weeks or 1 month (RR = 1.40; 95% CI, 1.20 to 1.62; *P* < 0.0001) (Fig. 8). The results showed that the short-term effect of herbal bath may be more obvious than the long-term effect in the treatment of UP.

Overall, compared with basic treatment (HD or HP) and adding combination with sham CHBT, clean hot water bath, or calamine lotion, all RCTs reported that Chinese herbal bath therapy plus basic treatment significantly relieved itching after 2 weeks to 3 months of treatment. However, the therapeutic effects of different herbal bath dosages is not very clear, and the long-term effect of CHBT for UP needs further study.

### Adverse events

Seven RCTs suggested that patients stop bathing if they felt unwell during the herbal bath treatment, but no adverse events were mentioned in any of the included RCTs. In addition, for safety reasons, 8 RCTs recommended adjusting the water temperature and bathing time according the patient’s physical condition, and 4 RCTs deemed that patients should not bathe when they are particularly hungry and full.

### Methodological quality

**Fig. 9:**
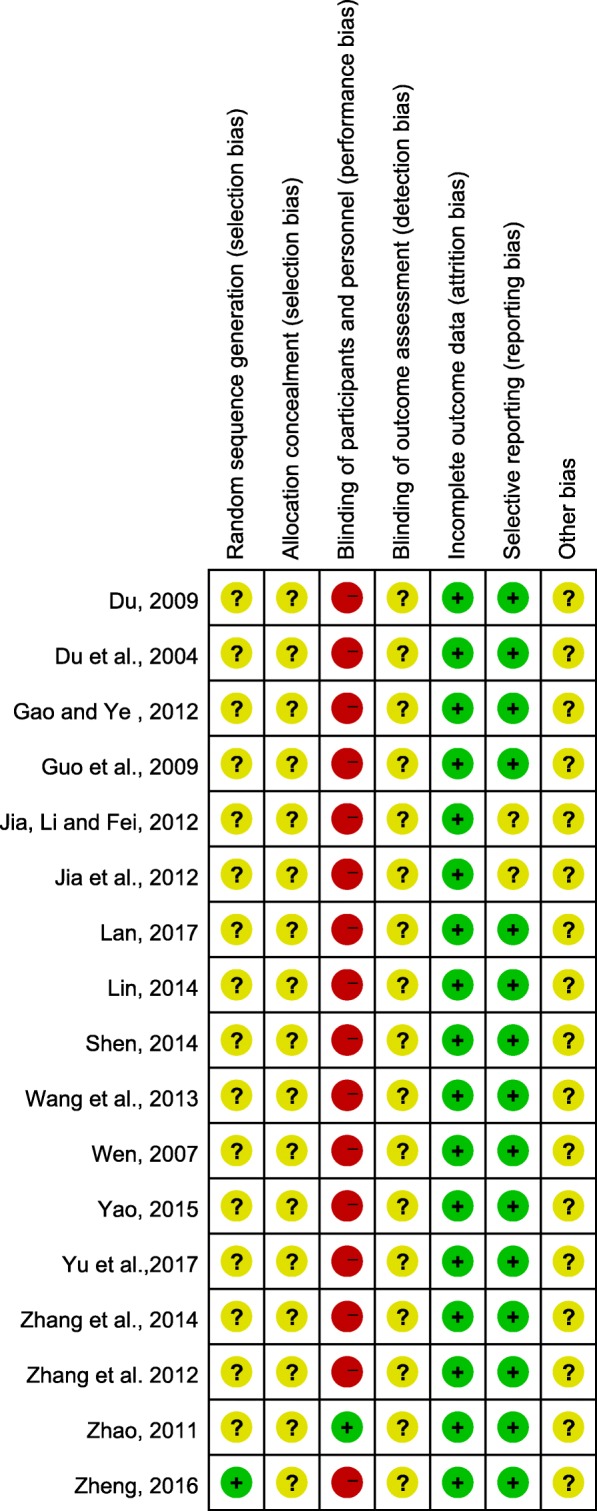
Risk of bias in the included randomized controlled trials

We assessed the risk of bias via the Cochrane Risk of Bias. According to the details of the results are presented in Fig. 9, the overall quality of the included trials was poor while two of them are moderate. All RCTs mentioned randomization, but only 1 article illustrated the specific grouping method using a random number table. None of the RCTs described allocation concealment or assessor blinding. In addition, only one trial used patient blinding. Meanwhile, a high risk of selective reporting could have existed in two trials.

## Discussion

Chinese herbal bath therapy is a traditional medicine treatment that involves pouring the decoction or extract of Chinese medicine into warm water and then bathing. The therapeutic method is easy to master and can be done by skilled patients themselves. Moreover, the herbal medicines used are not expensive and will not increase the financial burden of UP patients. In recent years, many clinical reports have shown that herbal bathing can effectively improve the symptoms of pruritus in patients with uraemia, and even assist in the treatment of uraemia [39–43]. This meta-analysis of 17 RCTs in 970 individuals also indicated that Chinese herbal bath therapy has a good adjuvant therapeutic effect on the treatment of UP. The results showed that itch relief of UP patients may be more significant with an appropriate increase in the frequency and duration of each bath. However, there is no evidence that the effect of herbal bath for UP is better in the long term than in the short term.

Despite the lack of interpretation about the biologic mechanisms of herbal bath therapy for uremic pruritus, the synergy between the efficacy of Chinese herbs and heating power may well be the basis for improving symptoms [9, 44]. First, some Chinese medicated herbs have an anti-pruritic effect on the skin. The antipruritic herbs, such as Fructus Kochiae and Lightyellow Sophora Root, are commonly used in the herbal bath and can be absorbed through the skin [38, 45]. Second, some Chinese herbs described in Table 2 and hot water bath itself can accelerate blood circulation and promote metabolism. The hot stimulation of an herbal bath can dilate local blood vessels, promote local and peripheral blood circulation and lymphatic circulation, and facilitate the effective ingredients in the bath liquid to play a role in the whole body through blood circulation. Third, some Chinese herbs and hot water bath can promote sweating to eliminate metabolic toxins. Studies have shown that the concentration of urea in sweat fluid can reach 5 to 50 times that in the serum. Similarly, the creatinine levels in sweat fluid have been shown to be high in patients with urinuria [46–48]. Therefore, it is important to exclude urea and creatinine by sweating when kidney function is impaired. Song and Ma found that haemodialysis combined with herbal bath had a significant effect on the sweat composition of patients, which can increase the excretion of potassium, uric acid, urea nitrogen, phosphorus and creatinine, and reduce the output of β2-microglobulin and parathyroid hormone in the sweat [49, 50]. In addition, several RCTs [33, 35, 51–53] have revealed that herbal bath therapy can reduce the level of serum creatinine and blood urea nitrogen in patients with uraemia. With the elimination of toxins, itchy symptoms can be effectively relieved.

No adverse reactions were reported in the included RCTs. As an external treatment, water temperature and bath time can be controlled, so the safety of Chinese herbal bath therapy is high. However, in traditional Chinese medicine, the hot herbal bath was not used on extremely weak patients due further depletion of body fluids and some nutrients because of perspiration in a hot bath. Therefore, it is important to have a clear assessment of the physical condition of UP patients before the herbal bath treatment [54].

Overall, CHBT is effective, safe, simple and inexpensive. In view of the above advantages of herbal bath for UP, we believe that normative clinical guidelines should be developed in future. To achieve the goal, some difficult problems need to be solved. For example, different dosages of CHBT (frequency, time, duration) and different herbs used may lead to different therapeutic effects. Which dosages are the better choices? Which herbs are more effective? Which UP patients do not suit CHBT? These aspects need to be further studied.

There are several limitations in our study that should be noted. First, the overall methodological quality of the included trials was poor based on the Cochrane handbook. In fact, there were some methodological flaws that might involve a high risk of bias in most of the RCTs. Only one study [35] reported the details of randomization, and no trial mentioned double blinding. In addition, these RCTs generally lacked careful follow-up, and the treatment courses of some trials were only 2 weeks. Therefore, the long-term efficacy and safety of CHBT for UP were not easily assessed. Second, the publication bias, which was caused by all the studies being published in China, may exaggerate the efficacy of CHBT to some extent. Third, the included RCTs were all single-centre studies, and the sample size of the experimental studies was relatively small, which made the research results lack reliability. Fourth, there were some discrepancies in the interventions of the control groups in the trials. Consequently, the efficacy of Chinese drug bath therapy for UP needs to be further confirmed by more well-designed, large-scale clinical trials.

## Conclusion

Chinese herbal bath therapy could be a complementary therapy to improve pruritic symptoms in uraemia patients. However, the methodological quality of these included trials was rated to be poor considering the risk of bias. More high-quality RCTs with rigorous designing are needed to further identify the efficacy and safety of CHBT for UP.

## Additional file

Additional file 1: Links of the 17 included articles. (DOCX 21 kb)

## Abbreviations

CBM: Chinese Biomedical database
CHBT: Chinese herbal bath therapy
CL: Calamine lotion
CNKI: China Network Knowledge Infrastructure
DRKS: Dirk R Kuypers score
HD: Haemodialysis
HP: Haemoperfusion
MD: Mean difference
OTCMT: Qther traditional Chinese medicine treatment
PRISMA: Preferred Reporting Items for Systematic Review and Meta-analysis
RCT: Random Controlled Trial
RR: Risk ratio
SD: Standard deviation
UP: Uremic pruritus
VAS: Visual analogue scale
VIP: Chinese Scientific Journal Database
