# Supplementary material for: Chinese herbal bath therapy for the treatment of uremic pruritus: meta-analysis of randomized controlled trials
Source: BMC Complement Altern Med. 2019 May 10;19:103. doi: 10.1186/s12906-019-2513-9 (PMC6509860; doi:10.1186/s12906-019-2513-9)
Supplement: Supplementary file 1 — Links of the 17 included articles. (DOCX 21 kb) [file 12906_2019_2513_MOESM1_ESM.docx]

Additional file 1: Links of the 17 included articles

1. Du W: Clinical observation on the treatment of uremia combined with pruritus of skin by Chinese herbal bath. Chinese Community Doctors 2009, 11(22):122.

URL: <http://kns.cnki.net/KCMS/detail/detail.aspx?dbcode=CJFQ&dbname=CJFD2009&filename=ZGSQ200922155&uid=WEEvREcwSlJHSldRa1FhdkJkVG1CTXc0WTk5alVDZG9Pb1d5OVpoUTZoTT0=$9A4hF_YAuvQ5obgVAqNKPCYcEjKensW4IQMovwHtwkF4VYPoHbKxJw!!&v=MDYwNDl1UnRGQ2poVWIvQlB5cllmN0c0SHRqT3JZNUFZWVI4ZVgxTHV4WVM3RGgxVDNxVHJXTTFGckNVUkxPZlo>=

2. Du MJ, Wen YW, Sun LH: Chinese herbal bath for treating 15 patients with uremic pruritus. Clinical Journal of Traditional Chinese Medicine 2004(02):126.

URL: <http://kns.cnki.net/KCMS/detail/detail.aspx?dbcode=CJFQ&dbname=CJFD2004&filename=AHLC200402025&uid=WEEvREcwSlJHSldRa1FhdkJkVG1CTXc0WTk5alVDZG9Pb1d5OVpoUTZoTT0=$9A4hF_YAuvQ5obgVAqNKPCYcEjKensW4IQMovwHtwkF4VYPoHbKxJw!!&v=MjY3MThSOGVYMUx1eFlTN0RoMVQzcVRyV00xRnJDVVJMT2ZadVJ0RkNqaFZMN0pKQ1hIYmJHNEh0WE1yWTlIWVk>=

3. Gao JM, Ye JY: Clinical observation of 21 patients of uremic pruritus treated by Chinese herbal bath. Clinical Focus 2012, 27(03):257-258.

URL: <http://kns.cnki.net/KCMS/detail/detail.aspx?dbcode=CJFQ&dbname=CJFD2012&filename=LCFC201203033&uid=WEEvREcwSlJHSldRa1FhdkJkVG1CTXc0WTk5alVDZG9Pb1d5OVpoUTZoTT0=$9A4hF_YAuvQ5obgVAqNKPCYcEjKensW4IQMovwHtwkF4VYPoHbKxJw!!&v=MDczMTJGQ2poV3IvT0tTN05iYkc0SDlQTXJJOUdaNFI4ZVgxTHV4WVM3RGgxVDNxVHJXTTFGckNVUkxPZlp1UnQ>=

4. Guo XL, Tan JC, Wang G: Clinical observation of skin pruritus in hemodialysis patients treated by traditional Chinese herbal bath. China Modern Medicine 2009, 16(07):84-85.

URL: <http://kns.cnki.net/KCMS/detail/detail.aspx?dbcode=CJFQ&dbname=CJFD2009&filename=ZGUD200907057&uid=WEEvREcwSlJHSldRa1FhdkJkVG1CTXc0WTk5alVDZG9Pb1d5OVpoUTZoTT0=$9A4hF_YAuvQ5obgVAqNKPCYcEjKensW4IQMovwHtwkF4VYPoHbKxJw!!&v=MjI4MTc0UjhlWDFMdXhZUzdEaDFUM3FUcldNMUZyQ1VSTE9mWnVSdEZDdmtVN3ZLUHlyZWFyRzRIdGpNcUk5QVk>=

5. Jia YL, Li XM, Du JH, Chang Y: Observation on curative effect of treating uremic pruritus with Shengshizhiyang Tang. Journal of Hebei Medical University 2012, 33(12):1434-1436.

URL:

<http://www.wanfangdata.com.cn/details/detail.do?_type=perio&id=hbykdxxb201212026>

6. Jia YL, Li XM, Fei YL: Effect of Shengshizhiyang Tang on uremia itchy skin. Journal of Hainan Medical University 2012, 18(05):646-647+650.

URL:

<http://www.wanfangdata.com.cn/details/detail.do?_type=perio&id=SciencePaper201309240000581587>

7. Lan SY: Analysis on the Effect of Chinese Herb Medicated Bath in the Treatment of Cutaneous Pruritus of Maintenance Hemodialysis. Guangming Tradit Chin Med 2017, 32(19):2791-2793.

URL:

<http://kns.cnki.net/KCMS/detail/detail.aspx?dbcode=CJFQ&dbname=CJFDLAST2017&filename=GMZY201719022&uid=WEEvREcwSlJHSldRa1FhdkJkVG1CTXc0WTk5alVDZG9Pb1d5OVpoUTZoTT0=$9A4hF_YAuvQ5obgVAqNKPCYcEjKensW4IQMovwHtwkF4VYPoHbKxJw!!&v=MDgyMTF0RkN2a1ZyL09JaURSZDdHNEg5Yk5wbzlIWm9SOGVYMUx1eFlTN0RoMVQzcVRyV00xRnJDVVJMT2ZadVI>=

8. Lin YF: Clinical efficacy and safety analysis of Shengshizhiyang Tang washing for treating patients with uremic pruritus. Chinese Journal of Traditional Medical Science and Technology 2014(z2):32-32.

URL:

<http://www.wanfangdata.com.cn/details/detail.do?_type=perio&id=zgzyykj2014z2041>

9. Shen H: Clinical observation on the effect of Chinese herbal bath on relieving pruritus in hemodialysis patients. World Health Digest 2014(25):285-286.

URL:

<http://www.wanfangdata.com.cn/details/detail.do?_type=perio&id=zwjkwz201425376>

10. Wang LY, Zhao JS, Luo M, wang FL, Yang M: Clinical observation of Chinese herbal bath for treating 27 patients with uremic pruritus. Chinese Medicine Modern Distance Education of China 2013(20):35-35.

URL:

<http://kns.cnki.net/KCMS/detail/detail.aspx?dbcode=CJFQ&dbname=CJFD2013&filename=ZZYY201320029&uid=WEEvREcwSlJHSldRa1FhdkJkVG1CTXc0WTk5alVDZG9Pb1d5OVpoUTZoTT0=$9A4hF_YAuvQ5obgVAqNKPCYcEjKensW4IQMovwHtwkF4VYPoHbKxJw!!&v=Mjg4ODBlWDFMdXhZUzdEaDFUM3FUcldNMUZyQ1VSTE9mWnVSdEZDdmtWYi9JUHpmU2Q3RzRIOUxPcjQ5SGJZUjg>=

11. Wen YW: Chinese herbal bath for treating 21 hemodialysis patients with uremic pruritus. Journal of Emergency in Traditional Chinese Medicine 2007(05):623.

URL:

<http://kns.cnki.net/KCMS/detail/detail.aspx?dbcode=CJFQ&dbname=CJFD2007&filename=ZYJZ200705085&uid=WEEvREcwSlJHSldRa1FhdkJkVG1CTXc0WTk5alVDZG9Pb1d5OVpoUTZoTT0=$9A4hF_YAuvQ5obgVAqNKPCYcEjKensW4IQMovwHtwkF4VYPoHbKxJw!!&v=MDMwMDJUQmRMRzRIdGJNcW85TllZUjhlWDFMdXhZUzdEaDFUM3FUcldNMUZyQ1VSTE9mWnVSdEZDdmtWYnZOUHo>=

12. Yao L: TCM formula granule washing for treating for 40 patients with uremic pruritus. China Pharmaceuticals 2015(24(B11)).

URL:

<http://www.cqvip.com/QK/83595X/2015B11/668034961.html>

13. Yu YN, Wang LL, Wu XB: Curative Effect Observation of Applying Dispelling Wind and Dampness Itching Medicated Bath Therapy for Uremic pruritus. Journal of New Chinese Medicine 2017(12):64-66.

URL:

<http://kns.cnki.net/KCMS/detail/detail.aspx?dbcode=CJFQ&dbname=CJFDLAST2018&filename=REND201712022&uid=WEEvREcwSlJHSldRa1FhdkJkVG1CTXc0WTk5alVDZG9Pb1d5OVpoUTZoTT0=$9A4hF_YAuvQ5obgVAqNKPCYcEjKensW4IQMovwHtwkF4VYPoHbKxJw!!&v=MjUxMjhadVJ0RkN2a1dyM0JOeWpGYXJHNEg5Yk5yWTlIWm9SOGVYMUx1eFlTN0RoMVQzcVRyV00xRnJDVVJMT2Y>=

14. Zhang CL, Yu GZ, Gui XQ, Huang CE, Zhang XW: Experience of nursing uremic pruritus with Chinese medicine formula granule. Chinese Medicine Modern Distance Education of China 2014, 12(20):128-129.

URL:

<http://kns.cnki.net/KCMS/detail/detail.aspx?dbcode=CJFQ&dbname=CJFD2014&filename=ZZYY201420082&uid=WEEvREcwSlJHSldRa1FhdkJkVG1CTXc0WTk5alVDZG9Pb1d5OVpoUTZoTT0=$9A4hF_YAuvQ5obgVAqNKPCYcEjKensW4IQMovwHtwkF4VYPoHbKxJw!!&v=MjA5MzBSdEZDdmtXN3pLUHpmU2Q3RzRIOVhPcjQ5TlpvUjhlWDFMdXhZUzdEaDFUM3FUcldNMUZyQ1VSTE9mWnU>=

15. Zhang YL, Zhu HF, Wei SZ, Li J: Clinical observation of Chinese herbal bath for treating 78 patients with uremic pruritus. World Health Digest 2012(41):382-383.

URL:

<http://www.wanfangdata.com.cn/details/detail.do?_type=perio&id=zwjkwz201241434>

16. Zhao H: Open Cou discharge turbidity dipping treatments for uremia itchy skin clinical research. Master. Nanjing University of Chinese Medicine; 2011.

URL: <http://kns.cnki.net/KCMS/detail/detail.aspx?dbcode=CMFD&dbname=CMFD2012&filename=1011221470.nh&uid=WEEvREcwSlJHSldRa1FhdkJkVG1CTXc0WTk5alVDZG9Pb1d5OVpoUTZoTT0=$9A4hF_YAuvQ5obgVAqNKPCYcEjKensW4IQMovwHtwkF4VYPoHbKxJw!!&v=Mjg2NzF0RkN2bFY3M0tWRjI2SDdHNkg5WExyNUViUElSOGVYMUx1eFlTN0RoMVQzcVRyV00xRnJDVVJMT2ZadVI>=

17. Zheng J: Observation on the curative effect of Yangxuezhiyang Mixture on uremia patients. Contmporary Medicine 2016(24):160-161.

URL: <http://kns.cnki.net/KCMS/detail/detail.aspx?dbcode=CJFQ&dbname=CJFDLAST2016&filename=DDYI201624113&uid=WEEvREcwSlJHSldRa1FhdkJkVG1CTXc0WTk5alVDZG9Pb1d5OVpoUTZoTT0=$9A4hF_YAuvQ5obgVAqNKPCYcEjKensW4IQMovwHtwkF4VYPoHbKxJw!!&v=MjQ4MTMzcVRyV00xRnJDVVJMT2ZadVJ0RkN2bVU3M09JU25TWjdHNEg5Zk9xNDVFWjRSOGVYMUx1eFlTN0RoMVQ>=
